# Biofilm Formation of *Staphylococcus aureus* under Food Heat Processing Conditions: First Report on CML Production within Biofilm

**DOI:** 10.1038/s41598-018-35558-2

**Published:** 2019-02-04

**Authors:** Jian Miao, Shiqi Lin, Thanapop Soteyome, Brian M. Peters, Yanmei Li, Huishan Chen, Jianyu Su, Lin Li, Bing Li, Zhenbo Xu, Mark E. Shirtliff, Janette M. harro

**Affiliations:** 1 Guangdong Province Key Laboratory for Green Processing of Natural Products and Product Safety, Guangzhou, 510640 P. R. China; 2 School of Food Science and Engineering, South China University of Technology, Guangzhou, 510640 China; 3 Home Economics Technology, Rajamangala University of Technology Phra Nakhon, Bangkok, Thailand; 4 Department of Clinical Pharmacy, College of Pharmacy, University of Tennessee Health Science Center, Memphis, TN 38163 USA; 5 Department of Haematology, Guangzhou Women and Children’s Medical Center, Guangzhou Medical University, Guangzhou, 510623 China; 6 Overseas Expertise Introduction Center for Discipline Innovation of Food Nutrition and Human Health (111 Center), Guangzhou, China; 7 Department of Microbial Pathogenesis, University of Maryland, Baltimore, 21201 USA

## Abstract

This study aimed to evaluate the *Staphylococcus aureus* biofilm formation and Nε-carboxymethyl-lysine generation ability under food heat processing conditions including pH (5.0–9.0), temperature (25 °C, 31 °C, 37 °C, 42 °C and 65 °C), NaCl concentration (10%, 15% and 20%, w/v) and glucose concentration (0.5%, 1%, 2%, 3%, 5%, 10%, w/v). *S. aureus* biofilm genetic character was obtained by PCR detecting *atl*, *ica* operon, *sasG* and *agr*. Biofilm biomass and metabolic activity were quantified with crystal violet and methyl thiazolyl tetrazolium staining methods. *S. aureus* biofilm was sensitive to food heat processing conditions with 37 °C, pH 7.0, 2% glucose concentration (w/v) and 10% NaCl concentration (w/v) were favorable conditions. Besides, free and bound Nε-carboxymethyl-lysine level in weak, moderate and strong biofilm were detected by optimized high performance liquid chromatography tandem mass spectrometry. Nε-carboxymethyl-lysine level in *S. aureus* biofilm possessed a significant gap between strong, moderate and weak biofilm strains. This investigation revealed the biological and chemical hazard of *Staphylococcus aureus* biofilm to food processing environment.

## Introduction

To date, thermal processes have been extensively employed in food technology^1–3^. Due to the aggregation of glucose, protein and lipid, Maillard reaction and lipid oxidation are promoted during heat treatment, which leads to browning, unpleasant flavors, even chemical hazard. According to World Health Organization (WHO), defined as poisonous and harmful substances in food to human body, food hazardous substances have been concerned over the sustainability^4–7^. As a pillar industry in China, the output value of food industry was 11.1 billion Yuan in 2016.

**Figure 1:**
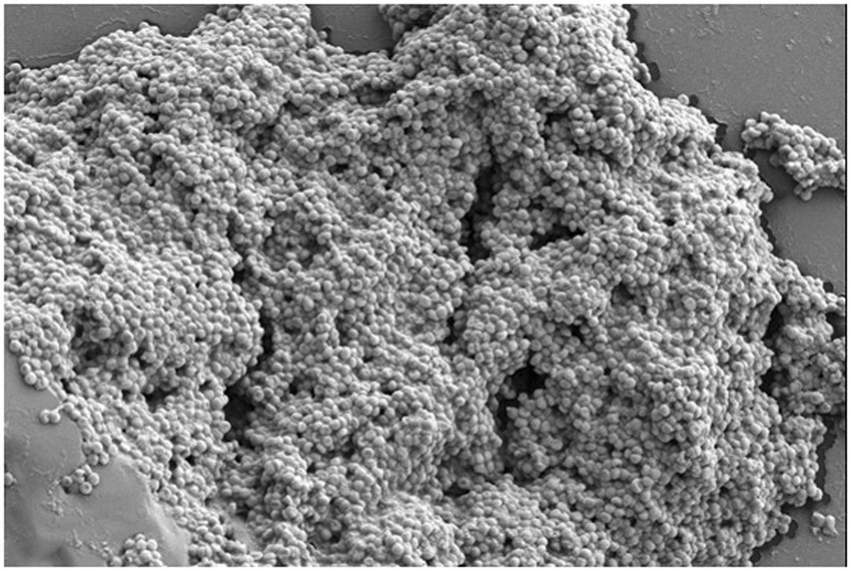
SEM image of *S. aureus* biofilm on the surface of un-washed stainless steel pipeline (5000x), Bar = 2 μm.

It’s recognized that over 80% microbial contamination was confirmed to be trigger by microbial biofilm formed on food processing equipment or raw material^8,9^. Among all bacteria retaining biofilm formation ability, *Staphylococcus aureus* (*S. aureus*) is a notorious foodborne pathogen capable of causing a spectrum acute food poisoning affairs and food processing equipment contamination. Considerable foodborne outbreaks caused by *S. aureus* were frequently reported over the years. From 1998 to 2008, 87% vomiting cases were led by *S. aureus* outbreaks in USA^10–12^. In China mainland, according to a recent investigation from 2006–2015, microbiological hazard have been considered as main food safety issues in China, with the proportion of 61.3%^13^. Similarly, another investigation revealed that *S. aureus* related food poisoning entailed a total morbidity of 2,431 people, among which meat (65.71%) and cereal & oil (34.29%) were main contributors^14–16^. In food industry, contamination caused by *S. aureus* biofilm mainly distributes in the following three aspects: (a) raw materials, especially the frozen food and animal food^17^; (b) processing environment and equipment, including meat abattoir and pipeline^18^ (Fig. 1, *S. aureus* biofilm on PVC pipeline surface); and (c) commodity circulation^19–22^.

Biofilm is a common strategy employed by bacteria to survive in varied adverse environment conditions to develop into a community of cells^23^ encased by extracellular polymeric substances (EPS). For *S. aureus*, its biofilm development is conducted in four stages^24^: attachment, microcolony formation, maturation and detachment. EPS is one of the most vital substances that essential for cellular encasement and community function in different stages. In *Staphylococcus* species, the primary matrix components consists of polysaccharide, proteins and extracellular DNA (eDNA)^23^. The first extensively studied matrix component is the exopolysaccharide termed PIA (Polysaccharide Intercellular Adhesin), which is produced and secreted by the proteins encoded in the *ica* (intercellular adhesion) operon *icaADBC*^25^, which include a N-acteylglucosamine transferase (IcaA and IcaB), a predicted exporter (IcaC)^26,27^, and a deacetylase (IcaD)^28,29^. The *ica* operon is important for biofilm formation in *S. aureus* with expression induced by environmental conditions including low oxygen, glucose, osmotic pressure and temperature^30,31^. Besides, some surface components were recognized contributing to adhesion in *S. aureus* biofilm including *Staphylococcus* protein A (Spa)^32^ and fibronectin binding protein (FnBP)^33^. Encoded by *atl*, Atl is a wall-anchored bifunctional peptidoglycan hydrolase responsible for cell separation following cell division^34,35^, which is required for biofilm development during early adhesion stage. In extracellular matrix, *S*. *aureus* surface protein G (SasG) contributes to the maturation phase of biofilm^36,37^. The eDNA is considered as an important structure component in bacterial biofilm^38^. The contribution could be retrospect in structural integrity maintenance, horizontal gene transfer and antagonism from antibiotics and the host immune system^39,40^. It has been proved that eDNA could bridge the energy barrier that separates the cell from the substratum, thereby facilitating irreversible adhesion^41^. Besides, eDNA also influences the hydrophobicity of bacterial cell surface and boost the tendency of a bacterium attachment^42–44^. Thus, in *S. aureus*, autolytic activity from a subpopulation of cells results in the release of eDNA that contributes to cell adhesion during biofilm maturation^45^. Quorum sensing (QS) system contributes greatly to cell density^46^ in cell communities encased in biofilm. Accessory gene regulator (*agr*) system is one of typical QS systems responsible for increased expression of various toxins and degradative exoenzymes, and decreased expression of several colonization factors, which resulted in degrading protein components of the *S. aureus* biofilm matrix^47–50^ and cell dispersion. The *agr*-mediated dispersion of *S. aureus* biofilm provides capacity of transfer in ideal environment such as food processing pipeline, which increased the difficulty in biofilm elimination.

The biological and chemical hazard of biofilm has been regarded as a public concern. Biofilm is easily formed under food processing conditions, especially heat processing with eutrophic condition, as well as chemical hazard within biofilm. Advanced glycation end products (AGEs) form in food during heating (especially in dry heat) and cause oxidative stress and chronic inflammation leading to increased risk for metabolic and cardiovascular events^51^. AGEs are products of the Maillard reaction, where sugar moieties react with proteins resulting in protein cross linking and product browning, together with formation of flavor and aroma compounds^52^. Since EPS consists of a matrix of polysaccharide and proteins with biological activity, AGEs could be accumulated within biofilm. Thus biofilm maintain the ability of both biological and chemical contamination during food processing. Nε-carboxymethyl-lysine (CML) is one of the most widely studied AGEs, and is frequently used as a marker for AGEs formation in food^53^, which is stable and exists both in free and bound form^54^. Free AGEs (glycated amino acids) and protein-bound AGEs (protein glycation adducts) may have different bioavailability and physiological effects^55^. CML in free AGEs (free CML) are much more bioavailable and harmful than CML in protein or peptide-bound AGEs (bound CML), since they are more easily released from a food system and absorbed into serum^56^. Studies have shown statistical association between accumulation of CML in the human body and diseases such as cardiovascular diseases, sarcopenia, and renal diseases^57^. Therefore, accurate quantification of free and bound CML is of great importance to reduce the intake of dietary CML and lower the risk of AGEs-related diseases^9,58–61^.

Food processing environments are easily continuously contaminated by microbes and harmful substances. Contamination of *S. aureus* is a vital public concern in consumers, to our knowledge, biofilm could generate CML under food heat processing while no study focused on it. This is the first report that biofilm formation character and CML level within biofilm under various food processing condition were investigated.

## Results

### *S. aureus* biofilm formation ability

As the result shown in Table 1, all three strains were identified as *S. aureus* and 4506, 120184 possessed the potential of strong biofilm formation ability (*ica*^+^, *atl*^+^, *sasG*^+^ and *agr*^+^). For biofilm phenotype, all three strains were submitted to CV staining and MTT staining assay. According to the PCR and staining results, strain 4506, 120184 and 10071 were considered maintaining strong, moderate and weak biofilm formation ability respectively. Though 120184 lacked *ic*a*A*, which was directly responsible for PIA synthesis^62^ in *S. aureus* biofilm, biofilm biomass and metabolic activity were still observed.

**Table 1:** Genetic character and biofilm formation ability of *S. aureus*.

Strain	Genes for detection and biofilm formation ability								Biomass and metabolic activity		Biofilm Formation Ability
	*16S rRNA*	*femA*	*icaA*	*icaBC*	*icaD*	*sasG*	*atl*	*agr*	OD_540nm_	OD_490nm_	
USA300*	+	+	+	+	+	+	+	+	NT	NT	
4506	+	+	+	+	+	+	+	+	4.68 ± 0.16	4.96 ± 0.10	Strong
120184	+	+	−	+	+	+	+	+	2.52 ± 0.33	2.06 ± 0.26	Moderate
10071	+	+	+	+	+	+	+	+	0.94 ± 0.28	1.11 ± 0.01	Weak

*USA300 strain was employed as positive control in *S. aureus* detection. NT = not tested.

**Table 2:** Primers for *S. aureus* identification and biofilm-related genes.

Primer	Target	Sequence (5′-3′)	*T*_*m*_ (°C)	Amplicon (bp)
*icaA*-F	*icaA*	TCTCTTGCAGGAGCAATCAA	56	188
*icaA*-R		TCAGGCACTAACATCCAGCA		
*icaBC*-F	*icaBC*	ATGGTCAAGCCCAGACAGAG	52	1188
*icaBC*-R		GCACGTAAATATACGAGTTA		
*icaD*-F	*icaD*	ATGGTCAAGCCCAGACAGAG	56	198
*icaD*-R		CGTGTTTTCAACATTTAATGCAA		
*atl*-F	*atl*	ACACCACGATTAGCAGAC	55	432
*atl*-R		AGCTCCGACAGATTACTT		
*sasG*-F	*sasG*	CGCTGATCAGAGATAAGAAAGGACCGG	50	1000
*sasG*-R		CGCTGATCATTAATTCTTTCTTCTACGAG		
*agr*-F	*agr*	GTGCCATGGGAAATCACTCCTTCC	52	976
*agr*-R		TGGTACCTCAACTTCATCCATTATG		
*16S rRNA*-F	*16S rRNA*	GGACTGTTATATGGCCTTTT	50	542
*16S rRNA*-R		GAGCCGTTCTTATGGACCT		
*femA*-F	*femA*	AAAGCTTGCTGAAGGTTATG	55	823
*femA*-R		TTCTTCTTGTAGACGTTTAC		

*16S rRNA* and *femA* were specifically designed for the screening of Staphylococci and *S. aureus* while 4 gene clusters including *ica*, *atl*, *sasG* and *agr* responsible for biofilm adhesion, maturation and dispersal were submitted to identification (Table 2). PCR reaction was conducted under program 94 °C for 5 min, followed by 94 °C 1 min, *T*_*m*_ 1 min, 72 °C 2 min for 30 cycles, 72 °C 7 min. Amplicons were stored under −20 °C.

### CML level in biofilm

**Figure 2:**
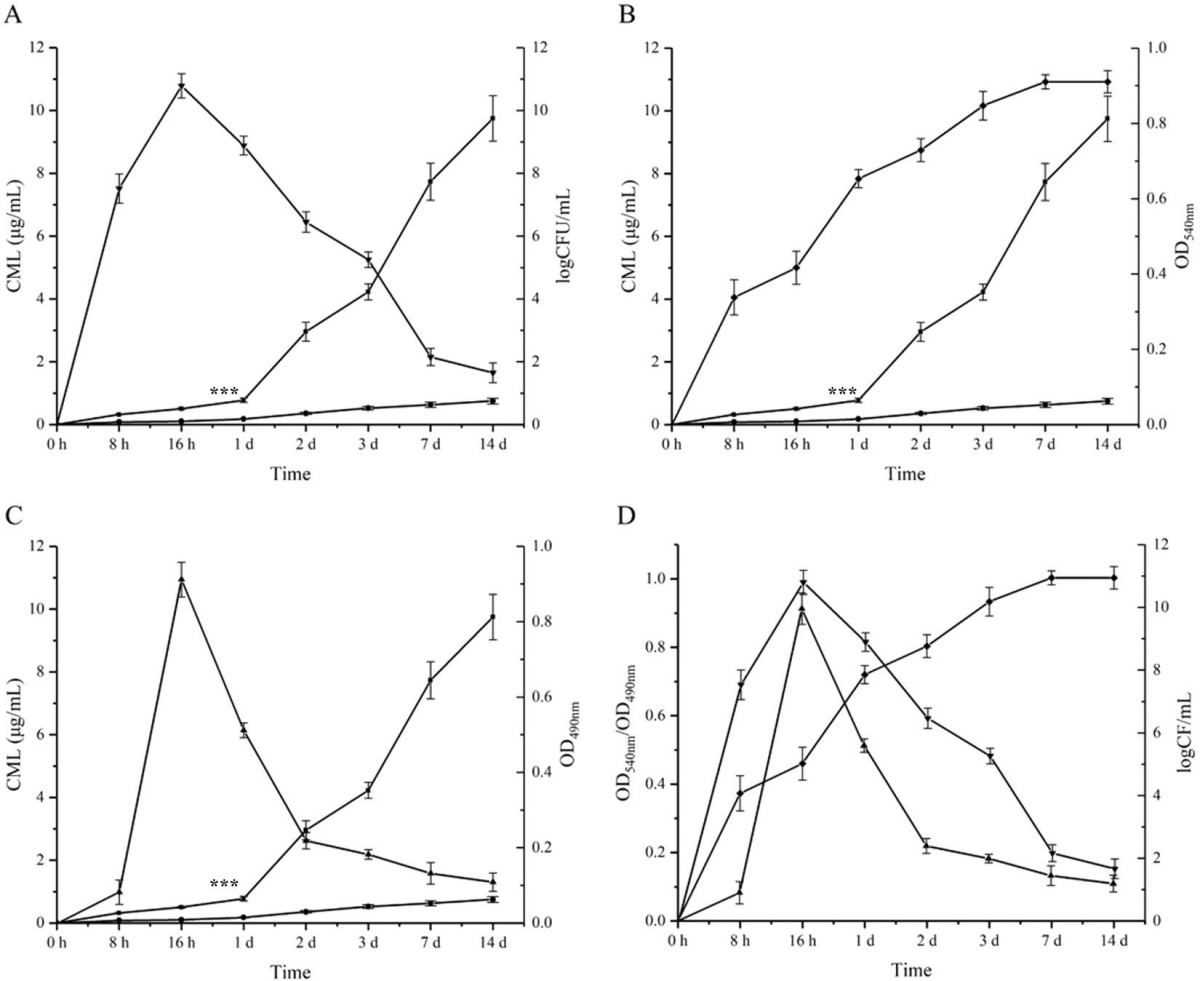
Free and bound CML level comparing with (**A**) live cells, (**B**) biomass, (**C**) metabolic activity. (**D**) Live cells, biomass and metabolic activity during incubation of *S. aureus* biofilm. ■ free CML, ● bound CML, ▼ CFU counting, ▲ MTT staining, ◆ CV staining. ***significantly different from bound CML (*P* < 0.001) as determined by one-way ANOVA.

For *S. aureus* strain 4506, free and bound CML level were quantified by HPLC-MS. Besides, CV and MTT staining assay as well as CFU counting were employed for quantification of biomass, metabolic activity and live cells in biofilm accordingly. In general, free CML level experienced a sharp increase since 24 h inoculation comparing to the stable bound CML level. For *S. aureus* biofilm, three stages were observed in this study accordingly (Fig. 2D). The number of live cells and biofilm metabolic activity reached peak at 16 h, with sharp decrease until 2 d. From 8 h to 1 d, free CML level increased with a slow rate while increased fast during 1 d-3 d when *S. aureus* biofilm turned to maturation. For bound CML level, as expected, the correlation between bound CML level and biofilm incubation time was observed, especially after biofilm adhesion stage (>24 h). Although the bound CML level was witnessed a steady increase, the concentration was far less than that of free CML (Fig. 2). Compare the results above, free and bound CML generation were significantly increased since maturation stage (>24 h). However, after the processing at 100 °C for 15 min, the bound CML was released from biofilm and was significantly higher than free CML (Fig. 2). It should be noticed that free CML showed the coordination with biomass of biofilm (Fig. 2B), instead of the number of live cells (Fig. 2A) and biofilm metabolic activity (Fig. 2C), which verified the assumption that CML was generated from the reaction of sugar moieties and proteins instead of direct cell synthesis and secretion.

**Figure 3:**
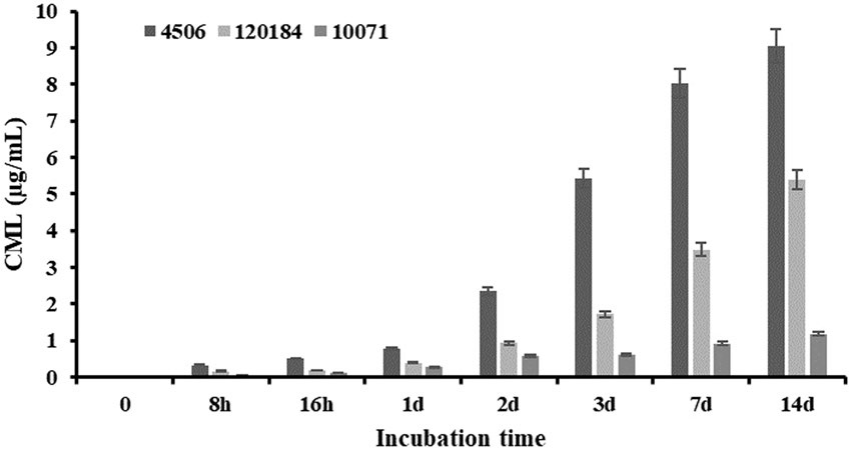
Bound CML level by biofilm formation ability.

According to several studies, the CML level in breast milk was lower than 0.150 μg/mL^63^, which was at same magnitude of free CML level in biofilm supernatant by strong biofilm formation ability strain 4506. However, there was a significant disparity of CML level in serum of 4.754 μg/mL^63^, which was at same magnitude of CML level since biofilm maturation started. Since the planktonic and supernatant were easy to remove and clearance while biofilm not, the CML in biofilm was accumulating until the heating process began. Thus, the bound CML was selected as a marker in the following study. As the results indicated (Fig. 3), a significant gap of bound CML level between strong, moderate and weak biofilm strains was observed. The bound CML level was significantly positive correlated with biofilm formation ability, in other words, biofilm biomass and metabolic activity.

### Influence of food processing conditions to *S. aureus* biofilm

**Figure 4:**
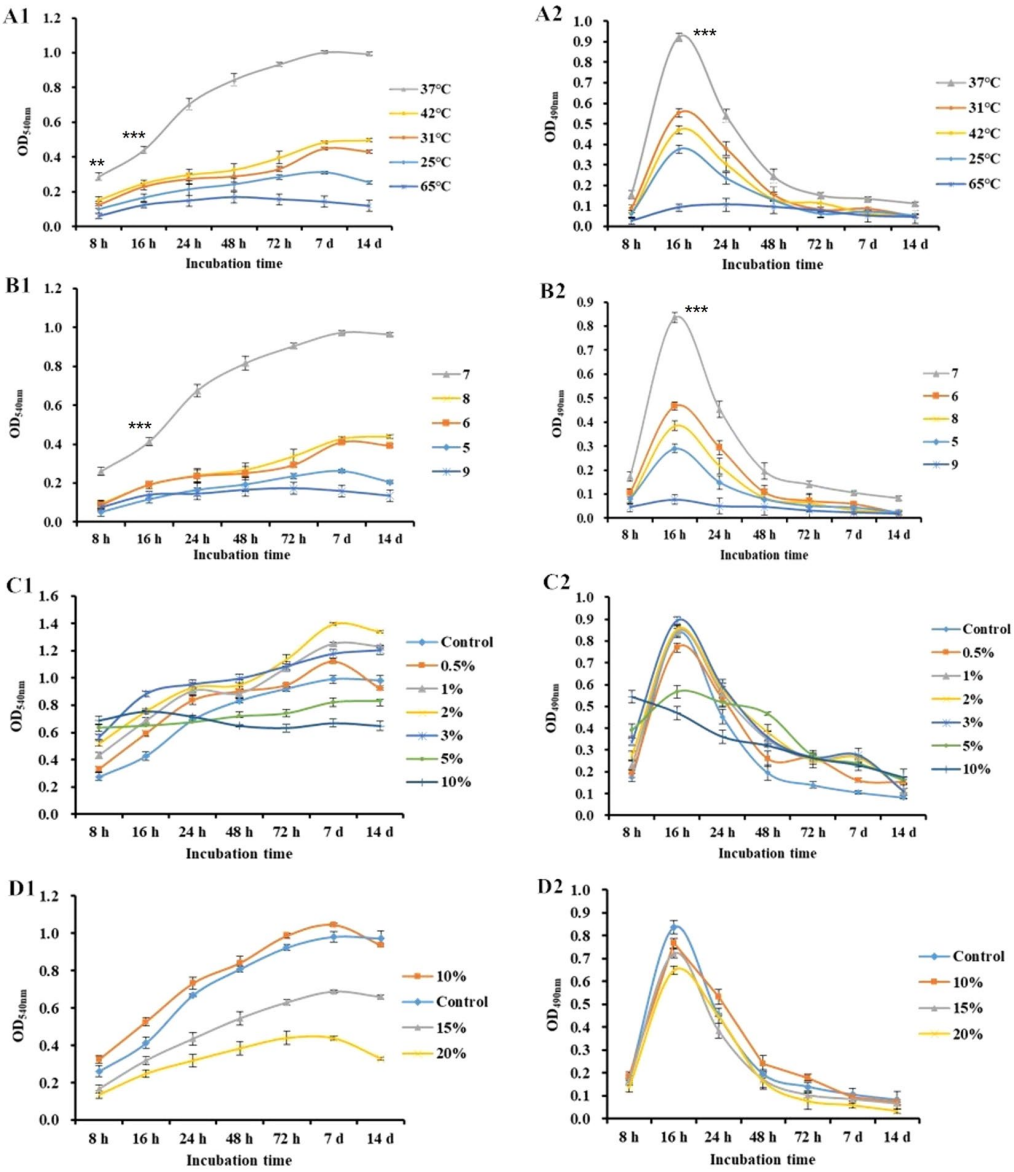
Influence of food processing environment to *S. aureus* biofilm biomass (series 1) and metabolic activity (series 2) in various (**A**) temperature, (**B**) pH, (**C**) glucose concentration (w/v) and (**D**) NaCl concentration (w/v). **/***significantly different from other columns (*P* < 0.01/*P* < 0.001) as determined by one-way ANOVA.

Four typical food processing condition were simulated including pH, eutrophy (glucose concentration), high-salinity (NaCl concentration) and temperature. *S. aureus* 4506 was selected in this part for its strong CML formation ability. As shown in result (Fig. 4), the suitable incubation temperature and pH were 37 °C and pH 7.0, with highest biomass and metabolic activity. Alkaline condition showed superior influence to the metabolic activity than acidic condition. Specifically, biofilm metabolic activity nearly lost under 65 °C and pH 9.0 incubation, which led a significant loss of biofilm biomass. For eutrophy condition, the biofilm metabolic activity showed a highly similar trend and peak except 5% and 10% glucose concentration. With the increase of glucose concentration, the peak of metabolic activity decreased sharply to 0.57 (5%, 16 h) and 0.55 (10%, 8 h). To 10% glucose concentration, the peak of metabolic activity occurred at 8 h, which was ahead of other concentration, and continuous declined. For biomass, biofilm showed an up-regulation with the increase of the glucose concentration at 8 h. A steady increase of biomass under 0.5%-3.0% glucose concentration was observed while a stable trend occurred to 5% and 10% glucose concentration and was less than 0% glucose concentration since 24 h. A hypotheses was conducted that *S. aureus* biofilm suffered earlier growth due to the increase of glucose concentration, but still need further study. A similar trend of metabolic activity in high-salinity condition was observed while 15% and 20% NaCl concentration showed a sharp decrease of biomass compare with control sample. The phenomenon was assumed to the osmotic pressure but still need further study.

## Discussion

To date, evidences of *ica*-independent pathways for biofilm forming have been confirmed^63^, and this phenomenon was observed frequently under various conditions^64,65^. In *ica*-independent pattern, extracellular matrix such as Spa and Bap act as compensatory substances for PIA in *S. aureus* biofilm^25^. Besides, FnBP, which was verified as a matrix regulated by autolysin and *sigB* in biofilm formation^26^ also contributes in *ica*-independent *S. aureus* biofilm. In this study, although strain 120184 was able to develop biofilm and maintain metabolic activity in biofilm, successfully without *icaA*, whether it was an *ica*-independent strain still need further investigation.

For CML level in *S. aureus* biofilm, according to our previous study, active metabolism was conducted inside the biofilm for colony proliferation, eDNA and EPS synthesis, as well as their secretion in maturation stage^65^, which may lead a dramatic increasement of CML level. However, there’s no evidences indicating that the accumulation of CML was accompanied by the metabolic activity of *S. aureus* biofilm. In fact, the number of live cells and biofilm metabolic activity decrease sharply while biomass of biofilm experienced a great increase since both free and bound CML came to accumulate in a rapid velocity (1 d, see Fig. 2). *S. aureus* biofilm turned to form a three-dimensional space structure containing eDNA, skeleton proteins, surface proteins and differentiated cells from maturation stage to maintain the stability, antagonize exogenous disturbances and prepare for the detachment and dispersal. The shield structure maintained integrality of biofilm, in which the bioactive proteins and polysaccharides were in close proximity to each other. With the heat processing of 100 °C for 15 min, CML accumulated inside the biofilm duo to protein and polysaccharide interactions while planktonic cells not, which illustrates a significant gap between free and bound CML level.

In food industry, most of the foodborne pathogens possess the ability of escaping from routine sterilization (such as high temperature and pressure, mechanical flushing) by forming biofilm, especially matured biofilm. Moreover, food components provides a eutrophic environment for biofilm formation, as well as the metabolism inside the biofilm, which leads an accumulation of metabolite such as CML. The most active stage in biofilm formation was 16 h, at which biofilm was at adhesion stage with the rapid proliferation and metabolism inside. The optimal environment for *S. aureus* biofilm formation was 37 °C, pH 7.0, with the glucose and NaCl concentration of 2% and 10% respectively. Although biofilm maintained resistance to adverse environment^66^, our study illustrated that *S. aureus* biofilm was sensitive to various temperature, pH and osmotic pressure conditions, which is commonly exist in food processing industry. Specifically, processing conditions of some typical staple food and snacks, such as noodle, rice cake and preserved fruit^67–70^, are under thermal, eutrophic and hyper osmosis processing conditions, which may accumulate *S. aureus* biofilm formation and CML level. Though daily cleansing is supposed to be conducted with regular mechanical cleansing methods (brush and jet cleaning with high temperature and high pressure), evidence has indicated the low efficiency in biofilm removal^71^, especially matured biofilm. The remnant nidus leads continuous formation and dispersion of biofilm, thus triggers sustained contamination in processing equipments, which caused the potential safety hazard.

During food heat processing, salt and glucose addition and pH adjustment are commonly conducted^72^ as well as the CML generation. Studies have been conducted to quantify CML level in food samples. But to our knowledge, there’s no study has been carried out for CML level quantification and influence of food processing conditions in foodborne pathogen *S. aureus* biofilm. Overall, current results indicated that *S. aureus* biofilm was sensitive to various temperature, pH and osmotic pressure conditions, with 37 °C, pH 7.0, 2% glucose concentration (w/v) and 10% NaCl concentration (w/v) were favorable conditions for biofilm formation. Besides, CML level in *S. aureus* biofilm possessed a significant gap between strong, moderate and weak biofilm strains. Based on the findings in current study, it’s supposed to raise the concern of *S. aureus* biofilm for its biological and chemical contamination in food heat processing condition.

## Material and Methods

### Strains and references

Three *S. aureus* strains (4506, 10071, 120184) were isolated from food samples. Standard strain USA300 and CML with 98% purity (Toronto Research Chemicals, Canada) were employed as reference substance.

### Identification and screening of *S. aureus*

Genomic DNA from *S. aureus* strains for PCR amplification were extracted from overnight tryptic soy broth (TSB) cultures at 37 °C with shaking. Culture was then performed according to the instructions of DNA extraction kit (Dongsheng Bio, Guangzhou) strictly. In short, harvested cells were subjected to the treatment of lysozyme, proteinase K and lysate orderly. Suspension was then purified after the removal of proteins and salts etc. The highly purified DNA was strictly stored under −20 °C.

### Biofilm formation and quantification

The overnight culture of *S. aureus* was diluted 1:100 into pre-warmed TSB and incubated at 37 °C until exponential growth began. The 200 μL exponential-phase culture was then inoculated into 6-well microtiter polystyrene plates (Corning, USA) for biofilm formation. After incubation for 48 h under 37 °C, the supernatants were removed and washed the plates three times with 200 μL sterile normal saline.

Cristal violet (CV) staining and AQ_ueous_ One Solution Cell Proliferation (MTT) Assay (Promega, USA) were employed for quantification of biofilm biomass and metabolic activity. Targeting to both viable and non-viable cells, CV is a basic dye that binds to negatively charged molecules present on both the surface of bacteria and the extracellular matrix of biofilm by measuring absorbance at 540 _nm_. MTT is a yellowish aqueous solution and yields a water insoluble violet-blue formazan if suffer reduction by dehydrogenases and reducing agents present in metabolically active cells. According to several investigations, MTT is mainly reduced with NAD(P)H-dependent cellular oxidoreductase activity (in cytoplasm) and dehydrogenases in active organelles^73^. Thus, biofilm metabolic activity has been shown to be proportional to production of formazan by measuring absorbance at 490_nm_^74^.

Besides, CFU counting was employed for free and bound CML quantification of 4506 strain as assistance. For biofilm biomass, 100 μL of 99% methanol was added to each well for a 15 min incubation. After CV staining (150 μL, 0.01%) for 15 min, washing twice with 200 μL sterile normal saline before re-eluting the CV dye with 100% ethanol and shake gently until all crystal violet is dissolved. For biofilm metabolic activity, staining the biofilm with MTT working solution (150 μL, 1:10 diluted) in dark for 2 h after removing loosely attached biofilm cells. Optical density (OD) represented biomass and metabolic activity under OD_540nm_ and OD_490nm_. All the operations above were proceeded under room temperature unless otherwise noted. The absorbance of an uninoculated well serves as a negative control (OD_c_) should be subtracted from the value of the inoculated wells. The following classification was applied for biofilm biomass and metabolic activity determination: no biofilm production (OD ≤ OD_c_), weak biofilm production (OD_c_ < OD ≤ 2OD_c_), moderate biofilm production (2OD_c_ < OD ≤ 4OD_c_) and strong biofilm production (4OD_c_ < OD)^75^.

### Preparation and pretreatment of *S. aureus* biofilm

*S. aureus* biofilm was incubated under 37 °C for certain time (8 h, 16 h, 1 d, 2 d, 3 d, 7 d and 14 d) before submitting to HPLC-MS analysis. Six-well polystyrene plate was slightly titled to remove the planktonic bacteria and liquids. Biofilm was collected into ultrapure water by scrapping with pipette tips after washed twice with ultrapure water. The solution of biofilm samples were sucked out and stored in sterile PEP tube under −4 °C for the subsequent test.

For free CML analysis, the biofilm sample was dissolved by heating process (100 °C, 15 min) which is common employed as food processing condition. The solution was then loaded into the conditioned ^18^C SPE column (2,000 mg/12 mL, Agela Technologies, China), which was then eluted with ultrapure water. The eluent was collected and filtered with nylon 0.22 μm membrane before submitting to HPLC-MS analysis. For bound CML analysis, modified pretreatment procedures was employed in this study based on Assar’s protocol^53^. Biofilm sample was dissolved by heating process (100 °C, 15 min), which was mixed with sodium borate buffer (0.5 M, pH 9.2) to a final concentration of 0.2 M. Sodium borohydride (2 M in 0.1 M NaOH) was added to reach a final concentration of 0.1 M, followed by vortexing for 30 s and stand for 4 h. Adding trichloroacetic acid (TCA, 60%, v/v) to a final concentration for 20%. After a 30 min standing, solution was vortexing for 15 min under 4 °C, 7000 rpm for protein extraction and repeated twice for purification. Extracted proteins and peptides were hydrolyzed in 6 M HCl at 110 °C for 24 h before blowing by Nitrogen Evaporators (N-EV, Organomation, USA) and re-dissolved by ultrapure water. HCl was then removed under vacuum and hydrolysate was purified by ^18^C SPE column, dried under vacuum and dissolved in 300 μL methanol-distilled water (1:9, v/v) prior to analysis by HPLC-MS. All the operations above were proceeded under room temperature unless otherwise noted.

### Quantification of CML by HPLC-MS

The HPLC-MS method for CML analysis has been well described in several studies^28,30^. In this study, HPLC (Waters 1525, Waters, USA) tandem single quadrupole mass spectrometer (Waters Micromass ZQ, Waters, USA) was employed for free and bound CML quantification. Atlantis ^18^C column (150 × 4.6 mm, 5 μm particle size, Waters, USA) was selected as separation chromatographic column for HPLC with a mixture of methanol-water-formic acid (10:90:0.1, v/v/v) was selected as mobile phase of 0.5 mL/min flow rate. Separated analytes were detected by the MS operated in electrospray ionization (ESI) positive mode with single ion recording (SIR). Significant MS operating parameters were as follows: capillary voltage 3.0 kV, cone voltage 20 V, source temperature 100 °C and desolvation temperature 300 °C. The mass charge ratio of CML was 205 (M^+^). The matrix effects in biofilm samples (mixing four representative commercial brand at equal amounts) prepared by the pretreatment methods for free CML analysis and the optimal pretreatment conditions for bound CML analysis were investigated under the above HPLC-MS conditions. All of these prepared biofilm samples had no significantly matrix effects (*P* > 0.05, data not shown). The injection volume was 10 μL with a retention time of CML 3.3 min. The mass spectrum was calibrated with CML standard substance at levels of 0.5 μg/mL, 1 μg/mL, 10 μg/mL, 50 μg/mL and 100 μg/mL to obtain standard curve. Analytes were quantified by reference to an external standard calibration curve.

### Influence of food processing conditions to *S. aureus* biofilm

To investigate the influence of food processing environment to *S. aureus* biofilm, strain 4506 was submitted to biofilm incubation under simulated typical food processing environment including pH (5.0, 6,0, 7.0 and 8.0), glucose (0.5%, 1.0%, 2.0%, 3.0%, 5.0% and 10.0%, m/v), NaCl (10.0%, 15.0% and 20.0%, m/v) and temperature (25 °C, 31 °C, 37 °C, 42 °C and 65 °C) for 8 h, 16 h, 1 d, 2 d, 3 d, 7 d and 14 d respectively. Biofilm incubation condition and quantification of biomass and metabolic activity with CV and MTT staining assays were described above.

### Statistical Analysis

All experiments were performed in triplicate and reported as mean ± standard deviation (SD). Statistical treatments and analysis of variance (ANOVA) were carried out by Microsoft Excel (v2013), Origin Pro (v8.0) and SPSS (v19.0). Significant differences were defined as p < 0.05 and were based on one-way ANOVA.
